# Granulomatous Mastitis: An Autobiographical Case Report

**DOI:** 10.7759/cureus.66701

**Published:** 2024-08-12

**Authors:** Sneha N Shanbhag, Nandan M Shanbhag

**Affiliations:** 1 Department of Internal Medicine, Dubai Physiotherapy and Rehabilitation Centre, Dubai, ARE; 2 College of Medicine and Health Sciences, United Arab Emirates University, Al Ain, ARE; 3 Department of Radiation Oncology, Tawam Hospital, Al Ain, ARE; 4 Department of Palliative Care, Tawam Hospital, Al Ain, ARE

**Keywords:** idiopathic granulomatous mastitis (igm), breast inflammation, patient-centered care, diagnostic challenges, granulomatous mastitis

## Abstract

Granulomatous mastitis (GM) is a rare, benign inflammatory breast disease that predominantly affects women of childbearing age and often mimics breast carcinoma. The diagnosis requires histopathological examination due to nonspecific imaging findings. Treatment includes antibiotics, corticosteroids, and surgery, but no standardized protocols exist.

This autobiographical case report describes a 34-year-old woman with a tender breast lump following trauma, initially misdiagnosed as a simple abscess. Despite incision and drainage, she developed erythema nodosum, persistent fever, and arthritis, which responded to corticosteroids. Further investigation, including an ultrasound-guided biopsy and MRI, confirmed GM. Recurrent symptoms were managed with prednisolone and doxycycline, leading to significant improvement.

This case report aims to highlight the diagnostic challenges associated with GM, emphasizing the necessity for a detailed histopathological examination to achieve an accurate diagnosis. It also brings attention to the significant emotional impact on patients facing a rare and complex diagnosis. By presenting this case, we aim to highlight the critical importance of a comprehensive and multidisciplinary approach to patient care in managing GM effectively.

## Introduction

Granulomatous mastitis (GM) is a rare, benign inflammatory breast disease primarily affecting women of childbearing age. It is characterized by the formation of granulomas within the breast lobules, leading to clinical symptoms that often mimic those of breast carcinoma. This resemblance to more serious conditions makes GM a challenging diagnosis, necessitating a thorough histopathological examination for accurate identification. The disease usually presents as a tender breast lump, typically occurring in women who have recently breastfed, further complicating the clinical picture due to its similarity to other breast conditions [1].

The etiology of GM remains largely unknown, though several potential causes have been proposed. Infections, particularly with *Corynebacterium* species such as *Corynebacterium kroppenstedtii*, have been implicated in some cases. Studies have demonstrated the presence of these organisms in affected tissues, suggesting an infectious component in the pathogenesis of GM. For instance, a review of 34 cases found an association between GM and corynebacteria infection, particularly *Corynebacterium kroppenstedtii*, which was identified in many cases [2]. There is also evidence suggesting a potential link between GM and occult tuberculosis (TB) infection, though this is rare [3]. GM has been proposed to have an autoimmune component, where the immune system mistakenly attacks the breast tissue, causing granuloma formation [4]. Hormonal influences are also suggested, as GM commonly affects women of childbearing age, and cases have been reported in women with drug-induced hyperprolactinemia, linking hormonal imbalances to the disease [5]. Furthermore, some reports suggest that GM may result from a chemical reaction associated with oral contraceptive pills, leading to granulomatous reactions in the breast tissue [6]. In many cases, GM is considered idiopathic, meaning no specific cause is identified, and it is diagnosed histologically by the presence of granulomas confined to the breast's lobules without any detectable infectious or other specific cause [7].

GM exhibits a distinct geographical distribution, with higher incidences reported in specific regions. A scoping review examining global cases from 2011 to 2020 found that Turkey, Iran, and China had the highest reported GM cases. This suggests that certain populations might be more predisposed to developing GM, potentially due to genetic, environmental, or socio-cultural factors [8]. Another study in New Zealand identified a significant number of GM cases associated with *Corynebacterium* infection, particularly among Maori and Pacific Islander women, highlighting a possible link between ethnicity and susceptibility [9]. Similarly, a 10-year review from a large inner-city county hospital in the United States noted a predominance of cases among Hispanic women, suggesting demographic influences on disease prevalence [10].

This autobiographical case report details the clinical journey of a 34-year-old woman with GM, highlighting the diagnostic challenges and treatment complexities associated with this rare condition. The objective is to enhance understanding of GM, elucidate effective management strategies, and demonstrate the importance of a multidisciplinary approach in patient care. By sharing this personal narrative, the report aims to contribute to the broader knowledge of GM and provide support to clinicians and patients facing this challenging diagnosis.

## Case presentation

In October 2020, a 34-year-old woman sustained trauma from her child's head bumping onto her left breast. She experienced a sharp pain that subsided with paracetamol. The following day, she discovered a painful lump in the same breast. She sought medical attention from a general surgeon, who recommended an ultrasound scan and blood tests. The ultrasound revealed edematous changes in the left breast without any fluid collection (Video 1), while the blood tests indicated an elevated erythrocyte sedimentation rate (ESR) of 49 mm/hr (normal <30 mm/hr; females) and C-reactive protein (CRP) of 118.7 mg/L (normal <10).

**Video 1:** Ultrasound of both breasts

**Figure 1:**
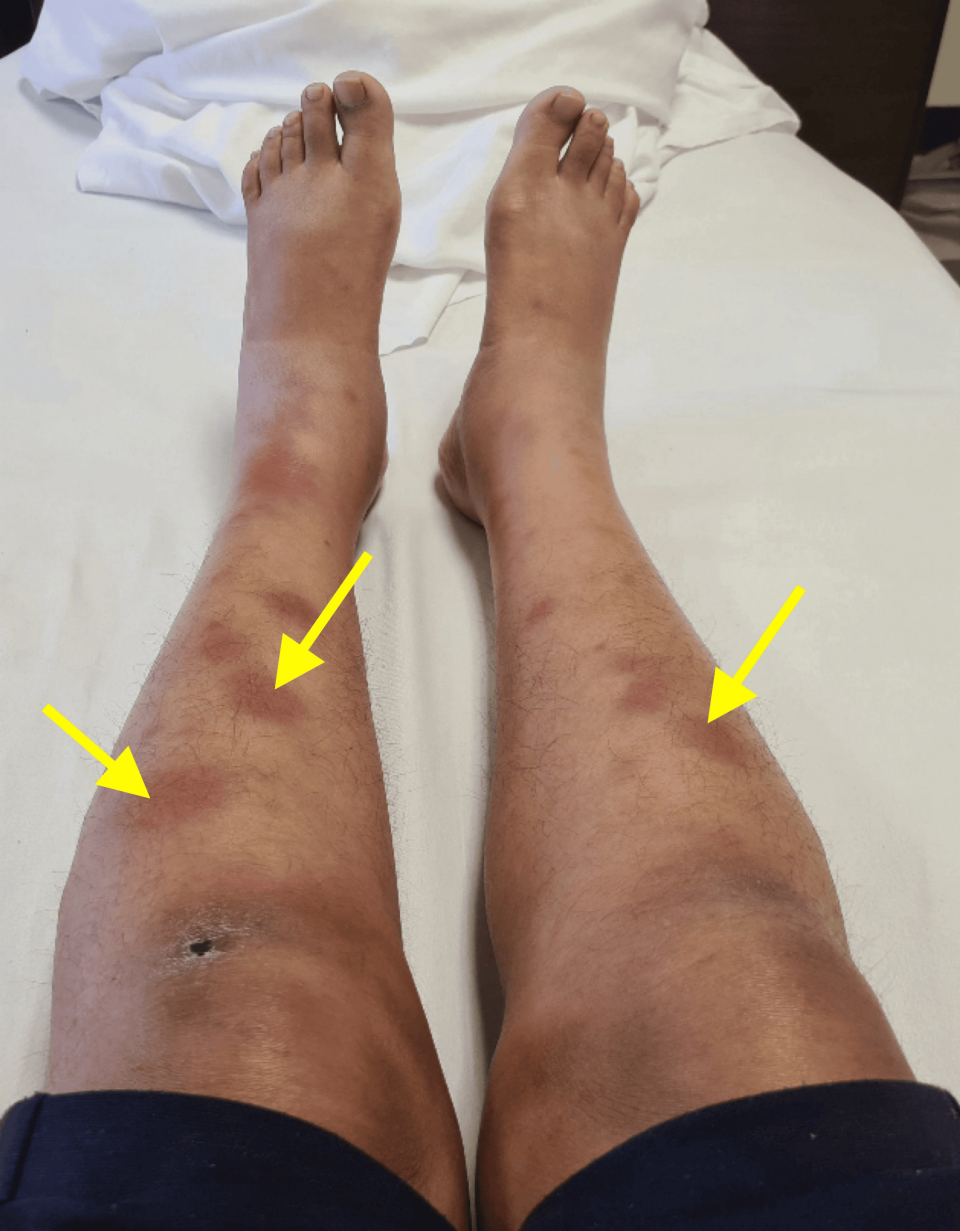
Erythematous nodules (yellow arrow) on both lower limbs

Over the next four days, the patient reported that the pain intensified significantly, becoming almost unbearable. The swelling in her breast progressively increased in size and tenderness, giving the sensation of pushing toward the surface and forming a noticeable lump. Upon physical examination, the area was red and warm to the touch, indicating inflammation. The lump was swollen and extremely tender. Palpation of the lump elicited a sharp pain response from the patient. The examining physician noted the firmness of the abscess under the skin and identified a small area suggesting potential spontaneous drainage. These physical findings highlighted the severity of the condition, necessitating prompt medical intervention. On November 12, 2020, the patient underwent an incision and drainage of the abscess under general anesthesia. The tissue obtained was sent for histopathological and culture analysis. She was prescribed amoxicillin and clavulanic acid (625 mg) twice daily for five days. By the fifth day, she developed reddish nodules on both lower limbs, painful joints, and a fever with chills. A complete blood count showed leukocytosis with a white blood cell count of 16 x 10^3^/µL (normal 4-10 x 10^3^/µL) and persistently high ESR of 53 mm/hr (normal <30 mm/hr; females) and CRP of 132.7 mg/L (normal <10) levels. She was diagnosed with erythema nodosum and admitted for further management (Figure 1).

Despite being started on intravenous ceftriaxone 2 gm twice daily for five days, her fever, arthritis, and nodular rash did not significantly improve after five days. The histopathological analysis of the breast tissue revealed multiple microabscesses and hematomas. The patient continued to exhibit persistent symptoms, including erythema nodosum, fever, and elevated levels of CRP, WBC, and ESR, indicating an ongoing inflammatory process. These systemic signs of inflammation necessitated the use of corticosteroids. Following complete abscess drainage, the patient was started on prednisolone and experienced immediate relief. She was discharged the next day with a prescription for 20 mg of prednisolone once daily for two weeks.

The breast abscess healed over the next two months, and the erythema nodosum subsided within one month. The prednisolone dose was gradually tapered to 10 mg and then to 5 mg, with complete cessation by the end of January 2021. Follow-up breast ultrasounds conducted twice during this period showed the presence of three cysts with no other significant changes, and the inflammatory markers had returned to normal levels.

**Figure 2:**
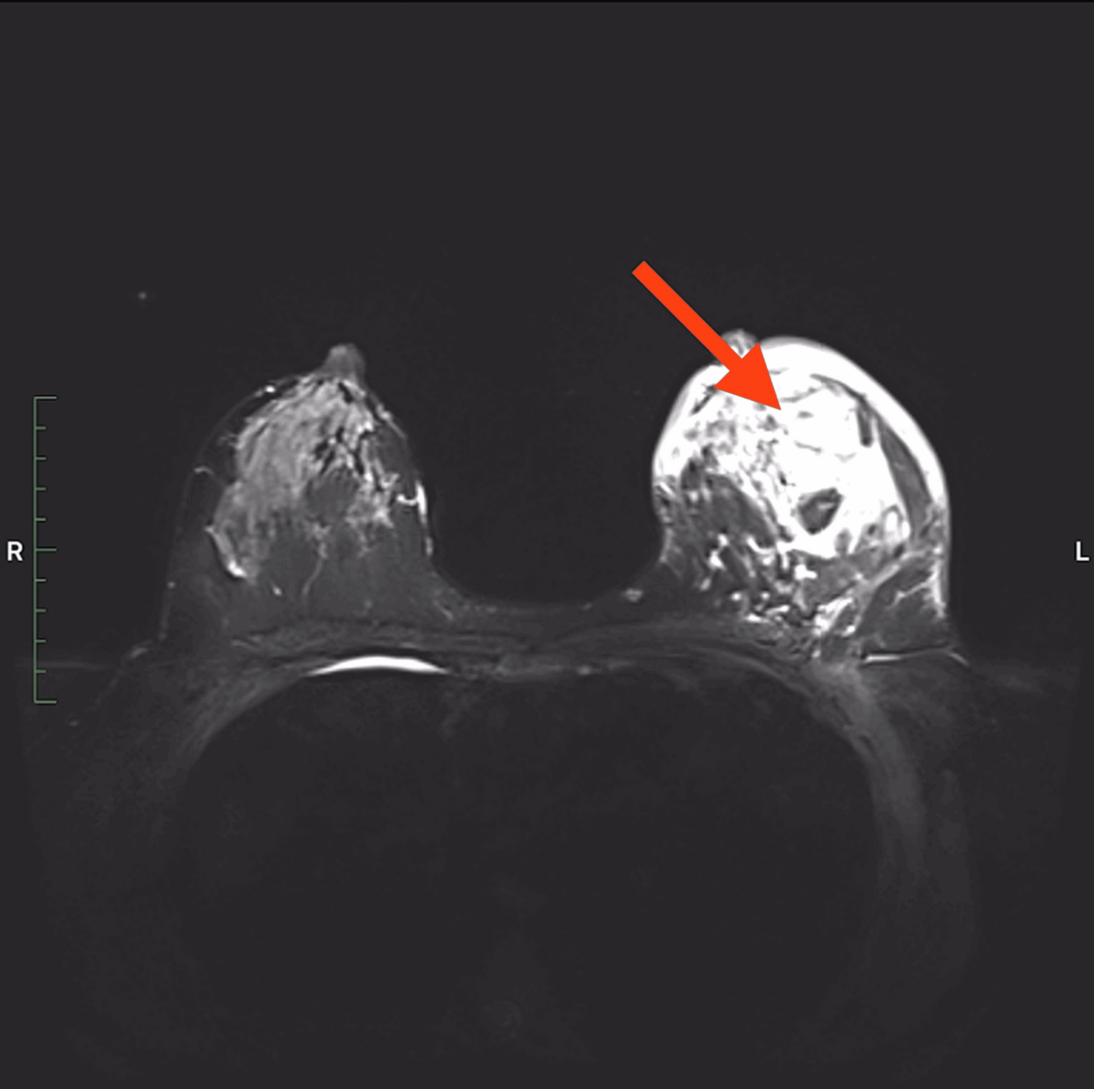
MRI of both breasts showing abscess in the left breast (red arrow) MRI: magnetic resonance imaging

**Figure 3:**
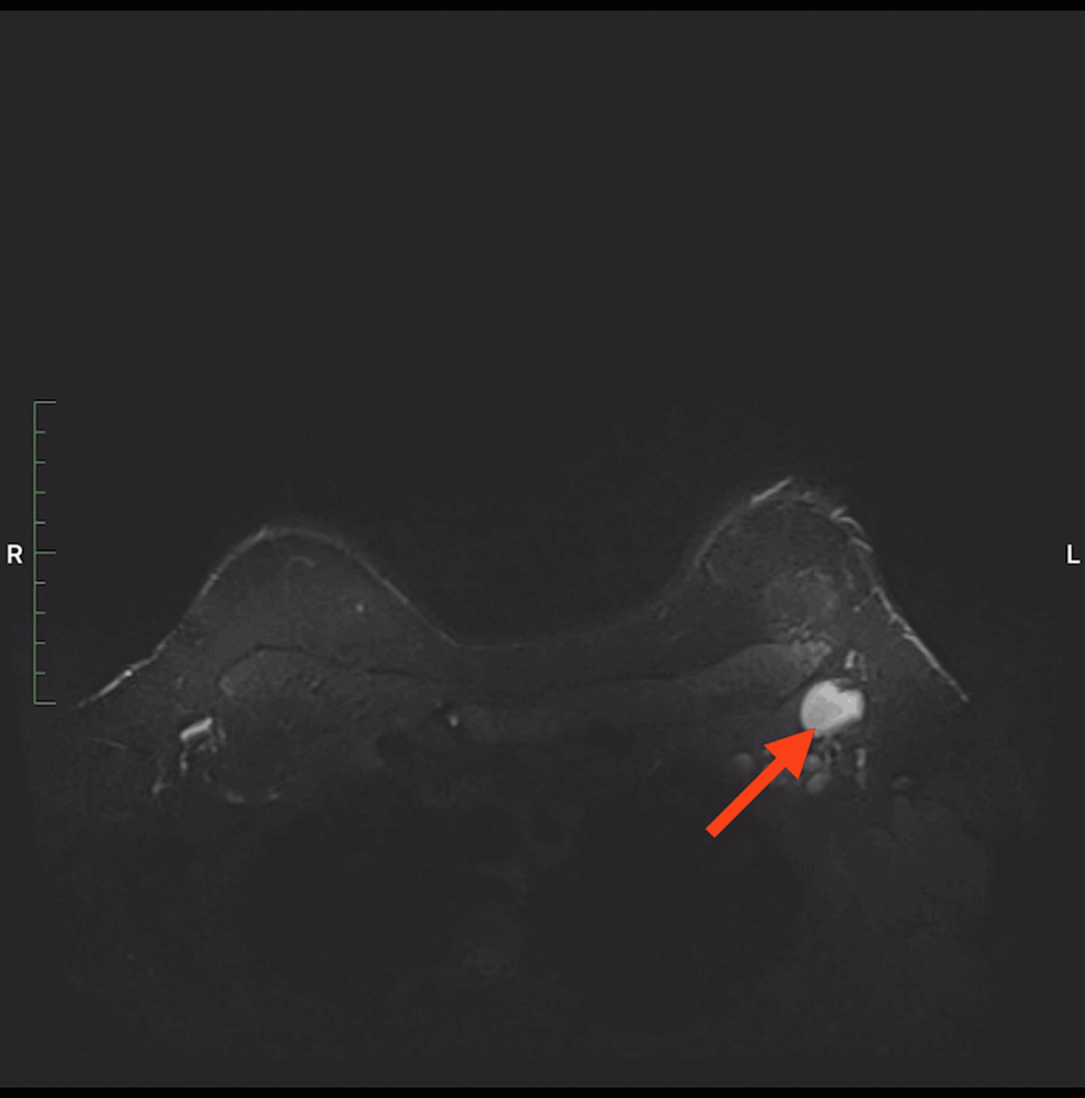
MRI of both breasts showing the left axillary node (red arrow) MRI: magnetic resonance imaging

The patient remained asymptomatic from January 2021 until October 2021. On October 29, she again felt a sharp pain in her left breast, which continued as a throbbing sensation. She consulted a breast surgeon, who suspected GM. An ultrasound scan revealed the same edematous changes noted previously, and oral cefuroxime 500 mg twice daily was started. Despite the treatment, the pain and swelling of the left breast persisted. On November 10, an ultrasound-guided breast biopsy confirmed GM, though an infective cause could not be ruled out. The MRI findings indicated evidence of mild right breast background glandular contrast uptake, measured at less than 25%. The left breast showed extensive nodular and non-mass-like high T2 and STIR signal intensity involving the upper and outer quadrants, extending to the retroareolar region. This area appeared hypointense on T1 imaging and exhibited a ring pattern of enhancement, which, along with restricted diffusion on diffusion-weighted imaging, supported the possibility of abscess formation. The overlying skin was markedly thickened, measuring 9.9 mm in the outer quadrant. The described lesion showed two foci with a noted curve pattern, raising intermediate concern. The right breast was normal, classified as Breast Imaging Reporting and Data System 2 (BI-RADS 2). The left breast, which showed lesions located in the upper and lower outer quadrants with a pattern of ring enhancement, restricted diffusion, and significant overlying skin thickening, suggested mastitis, favoring a diagnosis of GM with ipsilateral pathological axillary lymph nodes. The left breast was classified as BI-RADS 4B, indicating that a biopsy is warranted for further evaluation (Figures 2-3, Video 1) [11].

**Video 2:** MRI of both breasts MRI: magnetic resonance imaging

A biopsy was done, and samples were sent for polymerase chain reaction (PCR) identification of *Mycobacterium tuberculosis* DNA (TB PCR) and culture, which showed no growth. The presence of well-formed granulomas, multinucleated giant cells, lobulocentric inflammation, and microabscess formation without the identification of an infectious agent on special stains and cultures suggested an idiopathic etiology. Although *Corynebacterium kroppenstedtii* was not detected, the overall histopathological pattern supported a diagnosis of GM. Special stains and cultures were negative for TB and fungal infections.

On November 21, the patient developed erythema nodosum again, accompanied by fever with chills, multiple red nodules over both lower limbs, and severe arthritis involving the knees and ankles. She also had pitting pedal edema on both feet. The laboratory findings revealed several abnormalities indicative of inflammation and infection. Though the anti-nuclear antibody was negative, the patient's CRP level was significantly elevated at 137.20 mg/L. The WBC was also elevated at 15.10 x 10^3^/µL. Neutrophils were markedly high at 89.0%, while lymphocytes were low at 5.8%. The absolute neutrophil count was also elevated at 13.43 x 10^3^/µL, and the absolute lymphocyte count was low at 0.88 x 10^3^/µL (Table 1).

**Table 1:** Abnormal laboratory findings observed during the diagnosis and treatment of the GM case. GM: granulomatous mastitis, CRP: C-reactive protein, WBC: white blood cell, RBC: red blood cell, Hct: hematocrit, WNL: within normal limits, MCV: mean corpuscular volume, MCH: mean corpuscular hemoglobin, MCHC: mean corpuscular hemoglobin concentration, RDW: red cell distribution width, H: high, L: low

Test	Result	Unit	Normal range
CRP	137.2	mg/L	<10
Leucocyte count (WBC)	15.1	10^3^/uL	4.00-10.00
Erythrocyte count (RBC)	4.31	10^6^/uL	3.80-4.80
Hemoglobin (W. blood)	12.1	g/dL	12.0-15.0
Hct	36.8	%	36.1-46.0
MCV	85.4	fl	83.0-101.0
MCH	28.1	pg	27.0-32.0
MCHC	32.9	g/dL	31.50-34.50
Platelets	383	10^3^/uL	150-410
RDW	13.3	%	11.5-14.5
Neutrophils	89	%	40.0-75.0
Lymphocytes	5.8	%	20.0-45.0
Monocytes	5	%	2.0-10.0
Eosinophils	0	%	1.0-6.0
Basophils	0.2	%	0.0-2.0
Absolute neutrophils count	13.43	10^3^/uL	2.00-7.00
Absolute lymphocytes count	0.88	10^3^/uL	1.00-3.00
Absolute monocytes count	0.76	10^3^/uL	0.20-1.00
Absolute eosinophils count	0	10^3^/uL	0.02-0.50
Absolute basophils count	0.03	10^3^/uL	0.02-0.10

Prednisolone 20 mg was started on December 1, 2021, and the erythema nodosum subsided within five days. The breast continued to develop new abscesses, which the surgeon repeatedly aspirated without incisional drainage. The aspirate reported gram-positive bacilli, but the culture showed no sustained growth in 48 hours. By the end of December 2021, the TB PCR on the breast tissue was reported to be negative. Referred to an infectious disease specialist, the patient was suspected of having a slow-growing bacterial infection and was started on doxycycline 100 mg twice daily. The abscesses began to heal, and no new abscesses formed after two weeks. The pain and swelling in the left breast decreased over time. The steroid was tapered and stopped over two weeks, with cessation by the end of January 2022. The patient discontinued doxycycline in February 2022 after two months of treatment. The multiple drainage sites on the left breast were completely healed and closed by the end of April 2022. The patient has been on follow-up with her breast surgeon with no relapse and no medication and is disease-free.

## Discussion

The diagnostic process for GM can be particularly challenging when differentiating it from conditions such as tuberculous mastitis (TM), sarcoidosis, and breast carcinoma. In regions with a high prevalence of TB, GM can easily be misdiagnosed as TM due to overlapping clinical, radiological, and histological features. This diagnostic ambiguity can significantly complicate treatment plans and patient management [12].

GM typically presents with symptoms such as breast lumps, pain, and inflammation, which are also common in TM. The similarity in presentation necessitates a thorough diagnostic approach to rule out TB, as the presence of TB requires a different treatment regimen. Even when histopathological examinations show granulomatous inflammation, they do not definitively distinguish between idiopathic GM and TB mastitis [13]. TM is a rare form of extrapulmonary TB, typically presenting as a breast lump, sometimes accompanied by pain, nipple discharge, and axillary lymphadenopathy, accounting for less than 1% of all breast lesions and less than 3% of all cases of TB outside the lungs [14]. Imaging may show ill-defined masses and architectural distortion, similar to malignancies. Histologically, TM is characterized by granulomas with caseous necrosis, and acid-fast bacilli may be detected using Ziehl-Neelsen staining, with fine-needle aspiration cytology (FNAC) often revealing epithelioid cell granulomas and necrosis [15]. Treatment involves antitubercular therapy, which usually leads to resolution [15,16]. Unlike TM, GM shows non-caseating granulomas confined to the breast lobules, often associated with multinucleated giant cells and plasma cells, with no acid-fast bacilli presence. The diagnosis of GM is typically one of exclusion, requiring a thorough evaluation to rule out infectious causes like TB [17,18].

In my case, the diagnostic dilemma was heightened by my history of living in a region where TB is endemic [18]. Despite multiple tests, including histopathology, microbiological cultures, and PCR for *Mycobacterium tuberculosis*, the differentiation between GM and TB was not straightforward. Initial tests showed no growth, complicating the diagnosis further. This scenario is common, as studies have shown that TB can present similarly to other granulomatous diseases, leading to potential misdiagnosis [18]. The need for precise diagnostic tools is critical. FNAC coupled with PCR testing has been suggested as a reliable method to distinguish between TB and non-TB GM [19].

GM and breast carcinoma can present with similar clinical signs and imaging characteristics, making differentiation challenging. Clinically, both conditions often manifest as a palpable breast mass, which may be associated with pain, redness, and signs of inflammation such as erythema (redness due to increased blood flow), peau d'orange (a dimpled, orange peel-like skin appearance), and in some cases, erythema nodosum (painful, erythematous nodules on the legs) [20]. Imaging findings in both conditions are also nonspecific. Mammography and ultrasound can show ill-defined masses, and MRI may reveal non-mass enhancement with ring-like features, which suggest malignancy but can also be seen in GM [21,22]. However, histopathological examination is crucial for an accurate diagnosis. GM typically shows granulomatous inflammation centered on breast lobules with multinucleated giant cells, whereas breast carcinoma is characterized by malignant epithelial cells. A core needle biopsy or an excisional biopsy is often necessary to distinguish between these entities [23,24].

Treatment for GM is varied and remains a subject of debate due to the lack of standardized protocols and randomized controlled trials. Common therapeutic strategies include antibiotics, corticosteroids, immune modulators such as methotrexate, and surgical intervention. Corticosteroid therapy has shown efficacy in reducing inflammation and alleviating symptoms in some patients. However, the optimal treatment approach often depends on the individual case, with some patients responding well to medical management. In contrast, others may require surgical intervention to manage persistent or severe symptoms [25].

My journey with GM began in October 2020 when my child accidentally hit my left breast, causing sharp pain and a painful lump. The ensuing anxiety and fear were intense. As the pain worsened and an abscess formed, I underwent an incision and drainage procedure, which was a daunting experience. The diagnosis of erythema nodosum and subsequent hospital admission added to my emotional distress, with painful nodules and severe arthritis proving physically and mentally exhausting.

Starting on prednisolone brought some relief, but dealing with a chronic condition unresponsive to standard treatments left me feeling helpless. A brief period of calm followed, but in October 2021, the sharp pain in my left breast returned, reigniting my fears. Consulting a breast surgeon abroad and starting new medications while dealing with persistent pain and swelling was emotionally draining.

The recurrence of erythema nodosum and severe arthritis was devastating. Repeated aspirations for breast abscesses, ongoing medication, and fear of complications made me feel incredibly vulnerable. Despite these challenges, I found strength I didn’t know I had. The support of my family was invaluable, helping me cope emotionally. As my condition improved and I discontinued medications, I began to feel hopeful for the future.

My experience highlights the need for a multidisciplinary approach to ensure accurate diagnosis and treatment, avoiding unnecessary delays. This journey, marked by fear and frustration, has been managed through resilience and the unwavering support of my loved ones.

## Conclusions

In this case report, we described a 34-year-old female with GM characterized by persistent breast inflammation, erythema nodosum, and elevated inflammatory markers. Despite the lack of response to antibiotics, initiating corticosteroid therapy with oral prednisolone resulted in immediate symptomatic relief and significant clinical improvement. The histopathological examination revealed classic features of GM, including lobulocentric granulomas, multinucleated giant cells, and mixed inflammatory infiltrates. Special stains and cultures were negative for infectious agents, suggesting an idiopathic etiology.

This case illustrates the importance of considering GM in differential diagnoses of breast lesions and highlights the efficacy of corticosteroids in managing this condition. The diagnostic process posed significant challenges due to the need to exclude infectious causes and the rarity of the condition, which often mimics malignant breast disease. Additionally, the prolonged course of illness and multiple diagnostic procedures took an emotional toll on the patient, causing considerable anxiety and stress. Further studies are needed to better understand the underlying mechanisms and optimal treatment strategies for GM and to develop supportive measures to address the emotional impact on patients.
